# Knowledge of neonatal danger signs, care seeking practice and associated factors among postpartum mothers at public health facilities in Ambo town, Central Ethiopia

**DOI:** 10.1186/s13104-019-4583-7

**Published:** 2019-08-28

**Authors:** Gizachew Abdissa Bulto, Daniel Belema Fekene, Berhanu Ejara Moti, Getu Alemu Demissie, Keneni Berhanu Daka

**Affiliations:** 1 Department of Midwifery, College of Medicine and Health Sciences, Ambo University, Ambo, Ethiopia; 2 Department of Public Health, College of Medicine and Health Sciences, Ambo University, Ambo, Ethiopia

**Keywords:** Knowledge, Neonatal danger signs, Health care seeking practice, Ambo town

## Abstract

**Objective:**

Neonatal mortality has remained high in Ethiopia inspite of different efforts being undertaken to reduce this negative trend. Early detection of neonatal illnesses has an important step towards improving newborn survival. Toward this end, there is a need for postpartum mothers to be able to identify signs in neonates that signifies severe neonatal illnesses. There is limited information about the knowledge of post-partum mothers on NDSs and associated factors in the study area. This study aims to assess knowledge of NDSs, care-seeking practice and associated factors among postpartum mothers in Ambo town, Ethiopia, 2018. A systematic random sampling was employed to select respondents and data was collected through face-to-face interviews. Both bivariate and multivariable logistic regressions were utilized.

**Results:**

One-fifth 82 (20.3%) of postpartum mothers have good knowledge about NDSs. Only 60.5% of mothers whom their baby developed danger-sign sought medical care for their baby from health facility immediately. Mothers who have diploma/more education (AOR = 5.25, CI 1.48–18.59), whose current baby developed danger-signs (AOR = 3.18 CI 1.06–9.52), having PNC follow-up (AOR = 2.29, CI 1.24–4.24) and receiving counseling on newborn care after delivery (AOR = 1.78, CI 1.04–3.04) were factors associated with having good knowledge on NDSs. In this study the level of postpartum mother’s knowledge on NDSs and care-seeking practice were low.

## Introduction

The first 28 days of life the neonatal period are the most vulnerable time for a child’s survival. Globally, 2.6 million newborns died in 2016 or 7000 every day and Sub Saharan Africa accounts 38% of global newborn deaths. Half of all newborn deaths occurred in: India, Pakistan, Nigeria, Democratic Republic of Congo and Ethiopia. Studies showed that there are disparities across regions and countries to end preventable deaths of newborns to at least as low as 12 deaths per 1000 live births [1–3]. Majority of neonatal deaths (46.3%) occur within the first 24 h and 75% occur during the 1st week of life [4, 5]. In Ethiopia, neonatal-mortality ranges from 27.6 to 63/1000 live-births with 37 in 2011 and 29/1000 live-birth in 2016 [6–10].

Globally preterm birth, intrapartum related complications (birth asphyxia), infections and birth defects cause most neonatal deaths [5]. In Ethiopia neonatal sepsis, birth asphyxia and trauma, prematurity, congenital anomalies and acute respiratory infections were identified as the leading causes of neonatal deaths [6–8, 11, 12].

Neonates and young infants often present with non-specific symptoms and signs of severe illness. Neonatal danger sings (NDSs) signify the presence of clinical signs that would indicate high-risk of neonatal morbidity and mortality and the need for early therapeutic intervention [5, 13, 14]. In rural eastern Uganda newborns with danger-signs were at higher risk of death than those who weren’t [2]. Studies indicated that healthcare-seeking behavior of mothers was affected by their knowledge on NDSs which is important to recognize serious illnesses on the newborns. Maternal knowledge of NDSs and delays in deciding to seek-care were major contributors for neonatal mortality [15, 16]. From study done in four regions of Ethiopia only a third of women who reported neonatal illness had sought care [16]. In Tenta districts, only 41.3% of mothers sought medical care for neonatal danger signs [16].

Knowledge of mothers on NDSs varies in different studies; in Nepal Mean knowledge of 26.30%, Kenya 84.5% of mothers attending well-baby clinic had low level of knowledge, and in Ethiopia 50.6% in Mekelle city, 31.3% in Wolkite town, 43.7 in Aksum town, 50.3% in Chencha district, 18.2% in Gondar town and 21.7% in Woldia Hospital [17–23]. Studies indicated that different factors were affecting mother’s knowledge on NDSs. Lack of specificity of the clinical manifestations of various neonatal illnesses and delay in seeking care were resulting in huge mortality [17–19, 21, 24, 25].

In Ethiopia knowledge on NDSs were diverse, neonatal mortality rate is unacceptably high with little reduction; it is important to develop appropriate strategies and to reduce these unacceptably high death rates. Even though post-natal care (PNC) is one of the most important interventions for reduction of maternal and neonatal mortality it is not provided in a holistic manner including all PNC packages. The knowledge of postpartum mothers about NDSs, care-seeking practices for their newborn with danger-sign and associated factors in Ethiopia are limited, and there is no study done in the study area. Therefore the aim of this study is to assess knowledge on NDSs, care-seeking practice and associated factors among postpartum mothers in Ambo town.

## Main text

### Methods

#### Study design, area and period

Institutional based-descriptive cross-sectional study was conducted in public health facilities of Ambo town, from February 1st to March 30th 2018 in Ambo town, Oromia region. In Ambo town there are one University hospital, one General Hospital, 2 public health centers, and 13 private clinics. All postpartum mothers who brought their infant to immunization units for the first time at public Health Facilities in Ambo town and who were randomly selected during data collection were our study population. All temporary caregivers who brought infants for immunization were excluded.

#### Sample size and sampling procedure

Sample size was calculated using a single population proportion formula by considering: margin of error (5%), 95% confidence level 50.6% proportion of mothers who have good knowledge on NDSs taken from study done in Mekele town [17] and 5% non-response rate the final sample size becomes 404.

All three public health facilities providing immunization services were included in the study. Systematic random sampling technique was used to select 404 study subjects by using their immunization registration number as a sampling frame. The number of respondents were proportionally allocated based on their performance report and data was collected every 3rd interval, by using those health facilities 3 months’ performance of previous quarter [Ambo general hospital (461), Ambo health center (343) and Awaro health center (247)].

#### Measurements

Good knowledge of neonatal danger sign: Mothers who have mentioned three or more from the 11 key danger signs of neonate without prompt [17, 18, 20, 26].

The key neonatal danger signs include: Unable to feed/poor feeding, convulsion, respiratory rate of 60/more (fast breathing), severe chest in-drawing (difficulty in breathing), temperature of = 37.5 °C (fever), temperature = 35.5 °C (hypothermia), only moves when stimulated/not even when stimulated (weakness/lethargy), yellow soles (sign of jaundice), umbilicus redness or draining pus, skin boils, or eyes draining us (sign of local infection) and vomiting [14].

Care-seeking practice for NDSs: Those mothers who have taken their neonates to hospital or health center immediately after the neonate have developed danger signs.

#### Data collection technique, processing and analysis

Structured interview-administered Afan-Oromo version questionnaire adapted from reviewing different relevant similar literatures were used to collect data. A pretested questionnaire consisting of socio-demographic, maternal/obstetric health service utilizations, obstetric factors, knowledge of NDSs and care-seeking practices was used. Collected data were checked for completeness, coded and entered into EPI Info version 3.5.4 and exported to SPSS version 20 for analysis. Bivariate analysis was conducted primarily to identify those variables which are found to be associated with knowledge on NDS at p-value of < 0.2. Multivariable logistic regression analyses were conducted to identify associated factors at p-value of ≤ 0.05. Ethical clearance was obtained from ethics review committee of Ambo University, College of Medicine and Health Sciences.

### Results

#### Social demographic characteristics and health care utilization among respondents

A total of 404 mothers were interviewed during data collection making the response rate of 100%. In this study majority 371 (92%) of them were in the age group of 18–35 years old, 363 (89.9%) were married and 341 (84.2%) of them were from urban. One-third 122 (30.2%) of mothers attended secondary education, and 106 (26.2%) were government employees. Among all respondents 343 (84.9%) of them had ante-natal care (ANC) follow-up, 378 (93.6%) of them gave birth at health institution and 245 (60.6%) had PNC follow-ups (Table 1).

**Table 1:** Socio-demographic characteristics and maternal health care services utilizations among postpartum mothers attending immunization at health facilities in Ambo town, Central Ethiopia, 2018 (N = 404)

Variables	Frequency	Percentage (%)
Mothers age in year		
18–35	373	92.3
36–45	31	7.7
Mothers residence		
Urban	341	84.4
Rural	63	15.5
Mothers marital status		
Married	363	89.9
Single	15	3.7
Divorced/widowed	26	6.4
Mothers occupation		
Governmental employed	106	26.2
Self employed	77	19.1
House wife	87	21.5
Farmer	38	9.4
Merchant	49	12.1
Student	32	7.9
Other occupation	15	3.7
Mothers educational status		
Unable to read and write	44	10.9
Able to read and write	46	11.4
Primary school	82	20.3
Secondary school	122	30.2
Diploma and above	110	27.2
Mothers religion		
Orthodox	165	40.8
Muslim	28	6.9
Protestant	188	46.5
Other religion	23	5.7
Mother ethnicity		
Oromo	344	85.1
Amhara	55	13.6
Other ethnicity	5	1.2
Average family monthly income with Ethiopian birr		
< 1000	112	27.7
1001–2000	115	28.5
2001–3674	76	18.8
> 3675	101	25.0
Age of current baby in day		
0–28	88	21.8
> 29	316	78.2
Parity		
1	123	30.4
2–4	263	65.1
≥ 5	18	4.5
Attended ANC during latest pregnancy		
Yes	343	84.9
No	61	15.1
Number of ANC visits		
No ANC follow up	61	15.1
1st to 3rd visit	156	38.6
4th visit	187	46.3
Place of delivery		
Home	26	6.4
Hospital	218	54.0
Health center	160	39.6
No	111	27.5
Provided counseling on new born care after delivery before discharge		
Yes	137	33.9
No	267	66.1
Received PNC services		
Yes	245	60.6
No	159	39.4
Number of PNC visits		
No	159	39.4
Once	126	31.2
Twice	95	23.5
Three	24	5.9
Health education received during PNC follow up on		
Immunization	181	44.8
Family planning	161	39.9
Breast feeding and care	79	19.6
Neonatal danger sign	31	7.7
Care to ill neonates	8	2.0

#### Knowledge on neonatal danger signs

Mothers’ knowledge about NDSs was assessed; those who had mentioned more than or equal 3 general danger signs were considered as having good knowledge about NDSs. Only one-fifth 82 (20.3%) of postpartum mothers possess good-knowledge about NDSs (95% CI 16.1–24.8).

**Fig. 1:**
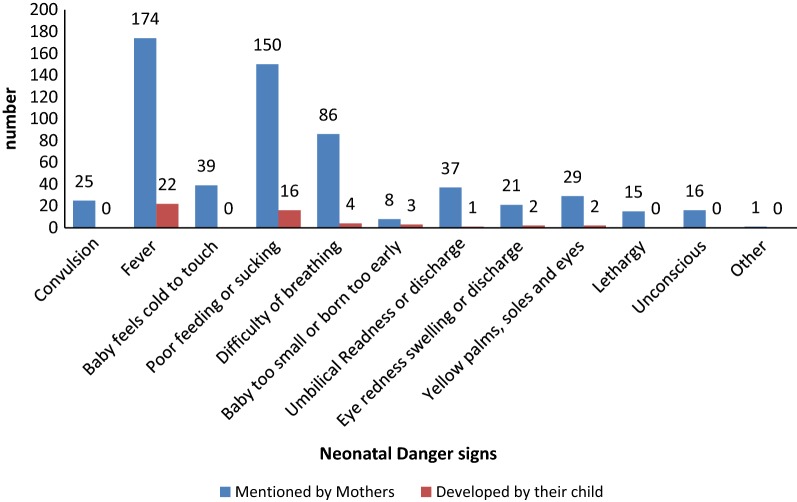
Distribution of neonatal danger signs and knowledge on neonatal danger signs among postpartum mothers attending immunization units at health facilities in Ambo town, Oromia, Ethiopia, 2018

Among all mothers, 282 (70.0%) of them were able to mention at least one NDSs and 121 (30.0%) don’t know any. Fever 174 (43.1%), poor feeding/sucking 150 (37.1%) and difficulty of breathing 86 (21.3%) were the commonly mentioned danger-signs. Major source of information for mothers were health-workers during delivery 155 (38.4%), from mass-media 34 (8.4%), health-worker during PNC 11 (2.7%) and 83 (20.3%) from other health workers. In 43 (10.2%) of mothers their current baby had developed one of those danger-signs of which 22 (5.4%) had fever and 16 (4.0%) of them had poor feeding/sucking. Among those whom their baby developed danger-sign 26 (60.5%) of them sought medical care from health facility immediately, 9 (20.9%) awaited and improved by itself and 8 (18.6.9%) sought medical care for their baby after it gets worsened (Fig. 1).

#### Factors associated with post-partum mothers’ knowledge on NDSs

On bivariate analysis mother’s education, occupation, average monthly income, source of information about NDSs, residence, ANC attendance, number of ANC visits, place of delivery, developed maternal danger sign, PNC follow-ups, new-born developed danger sign, counseled on newborn care before discharge after delivery and those who received education on danger sign were found to be associated with knowledge on NDSs at p-value of < 0.2.

On multivariable logistic regression analysis mother’s educational status, those who received PNC services, current newborn developed danger sign and those who were counseled on newborn care before discharge after delivery were factors that are found to be significantly associated with having good knowledge on NDSs.

In this study those mothers who have diploma/more education were five times more-likely to have good knowledge than those who are unable to read and write (AOR = 5.25, 95% CI 1.48–18.59) and those mothers whom their current baby developed danger signs were three times more-likely to have good knowledge than those who weren’t (AOR = 3.18 95% CI 1.06–9.52).

Current study also revealed that those mothers who had PNC follow-up were two times more-likely to have good knowledge than those who weren’t (AOR = 2.29, CI 1.24–4.24) and mothers who had received counseling on newborn-care after delivery were almost two-times more-likely to have good knowledge than those who weren’t (AOR = 1.78, CI 1.04–3.04) (Table 2).

**Table 2:** Factors associated with good knowledge about neonatal danger signs among postpartum mother’s attending immunization units in Ambo town, Oromia region, Ethiopia, 2018 (N = 404)

Variables	Knowledge on neonatal danger signs		COR with 95% CI	AOR with 95% CI	p value
	Good	Poor			
Mothers education					
Unable to read and write	3	41	1	1	0.015
Able to read and write	6	40	2.05 (0.48, 8.76)	1.91 (0.43, 8.39)	0.388
Primary school	16	66	3.31 (0.9, 12.07)	2.76 (0.73, 10.3)	0.131
Secondary school	21	101	2.84 (0.8, 10.04)	2.34 (0.64, 8.46)	0.195
Diploma and above	36	74	6.64 (1.92, 22.9)	*5.25* (*1.4*, *18.5*)	0.010*
Mothers residence					
Urban	77	264	3.38 (1.31, 8.73)		
Rural	5	58	1		
Attended ANC on last pregnancy					
Yes	78	265	4.19 (1.47, 11.9)		
No	4	57	1		
Number of ANC visits attend					
No follow-up	4	57	1		
1–2 times	10	39	3.65 (1.06, 12.4)		
3 times	29	78	5.29 (1.76, 15.9)		
4 times	39	148	3.75 (1.28, 10.9)		
Place of delivery					
Home delivery	2	24	1		
Health Institution	80	298	3.22 (0.74, 13.9)		
Received PNC follow up					
Yes	65	180	3.01 (1.69, 5.37)	2.29 (1.24, 4.24)	0.008*
No	17	142	1	1	
New born developed danger sign					
Yes	4	39	2.68 (0.93, 7.74)	3.18 (1.06, 9.52)	0.038*
No	78	283		1	
Counseled on new born care before discharge after delivery					
Yes	39	98	2.07 (1.26, 3.39)	1.78 (1.04, 3.04)	0.035*
No	43	224		1	
Received education on danger signs					
Yes	6	12	2.03 (0.74, 5.6)		
No	76	310	1		

*Statistically significant association at P-value of ≤ 0.05 with backward stepwise logistic regression

### Discussion

This study assessed the level of knowledge on NDSs and associated factors among postpartum-mothers at public health institutions in Ambo town. It was found that only 20.3% of postpartum-mothers were having good knowledge about NDSs (95% CI 16.1–24.8). Which is nearly inline with study conducted in Kenya (15.5%), Nepal (26.30%) and Gondar town (18.2%) mothers had good knowledge on neonatal danger signs [20, 22, 23]. But, the current study finding is lower-than studies done in Mekele (50.6%), Wolkite town (31.32%) and Chencha district Ethiopia (50.3%) of mothers have good knowledge about NDSs [17, 18, 21]. The possible reason for this variation might be due to the differences in health care services delivery in which information on danger-sign was not adequately disseminated to mothers both during antenatal and postnatal care, health care providers mainly focuses on care of mothers and neonates than giving health education on danger-signs in our study area.

Newborns with danger-signs were at higher risk of death those than who weren’t. Healthcare-seeking behavior of mothers for their newborn by recognizing those serious illnesses was important to avoid delays in making decision which contributes for neonatal mortality. In this study only 60.5% of mothers whom their baby developed danger-sign sought health care from health facility immediately. This finding was higher than study done in four regions of Ethiopia (1/3rd) and Tenta district (41.3%) women sought medical care for NDSs [27, 28]. The possible reason for this might be due to the variations in time, study setting, study population and cultural variations in which studies in four regions of Ethiopia and Tenta district were conducted in 2014 and 2015, were community-based, majority were from rural and there were cultural reasons of keeping neonates inside home; but in current study majority of them were from urban and it was conducted among those mothers who came for immunizing their babies.

In this study educational status of mothers were found to be significantly associated with their knowledge. Mothers with diploma or more education were five times more-likely to have good knowledge than those who are unable to read and write. This is in-line with studies done at Woldia general hospital, Gondar and Wolkite towns [18, 22, 24].

Maternal health services utilizations are also associated with higher knowledge of newborn danger signs. Current study revealed that those mothers who had PNC follow-up were two times more-likely to have good knowledge than those who weren’t and mothers who had received counseling on newborn care after delivery were almost two times more-likely to have good knowledge than those who weren’t. Incongruent with this study done in Mekele, Woldia hospital, Wolkite and North-west Ethiopia showed that PNC advice on NDSs, information about neonatal danger-signs, PNC attendance, access to television were factors for having good knowledge about NDSs [17, 18, 22, 24].

Mothers whom their current baby had developed danger signs were three times more-likely to have good knowledge than those who aren’t. The possible reason for this might be due to those mothers whom their current baby developed danger-signs were more-likely to seek information and care from health workers for their sick baby from where they could get awareness about NDSs.

### Conclusion

The level of postpartum mother’s knowledge on NDSs and care-seeking practice for their newborn with danger sign is low in the study area as knowing those general danger signs is important for early detection of serious illness and seeking health care for their child from health facilities. This study identified that those who had diploma/higher education, current baby developed danger-signs, PNC follow-ups and received counseling on newborn care after delivery before discharge were factors significantly associated with having good knowledge about NDSs.

Therefore provision of health education and counseling/advices for mothers after delivery and during PNC follows-ups on NDSs to improve their knowledge and health care-seeking behavior were the key areas of intervention.

## Limitations

Though the study tried to address knowledge and care-seeking practice of postpartum mothers who brought their children for 1st immunization it isn’t free of recall bias.

## Data Availability

Datasets used in current study are available from the corresponding author up-on request.

## Abbreviations

ANC: ante natal care
AOR: adjusted odds ratio
NDSs: neonatal danger signs
PNC: post natal care
